# The great mimicker: Pleural tuberculosis presents as nodular pleural thickening

**DOI:** 10.5339/qmj.2024.qitc.28

**Published:** 2024-03-26

**Authors:** Aasir M. Suliman, Muna Abuhejleh, Muhannad Salih, Mansoor Hameed

**Affiliations:** Pulmonology Department, Hamad General Hospital, Hamad Medical Corporation, Doha, Qatar Email: asuliman4@hamad.qa; Laboratory Medicine and Pathology Department, Hamad General Hospital, Hamad Medical Corporation, Doha, Qatar

**Keywords:** Tuberculosis, TB, pleural TB, pleural thickening

## Background

Pleural tuberculosis (TB) typically presents initially as pleural effusion or empyema and subsequently as pleural thickening. However, nodular pleural thickening, particularly in the absence of other parenchymal lung abnormalities, is atypical of pleural TB and is often a manifestation of malignancy.^1,2^

## Case Presentation

**Figure 1.**
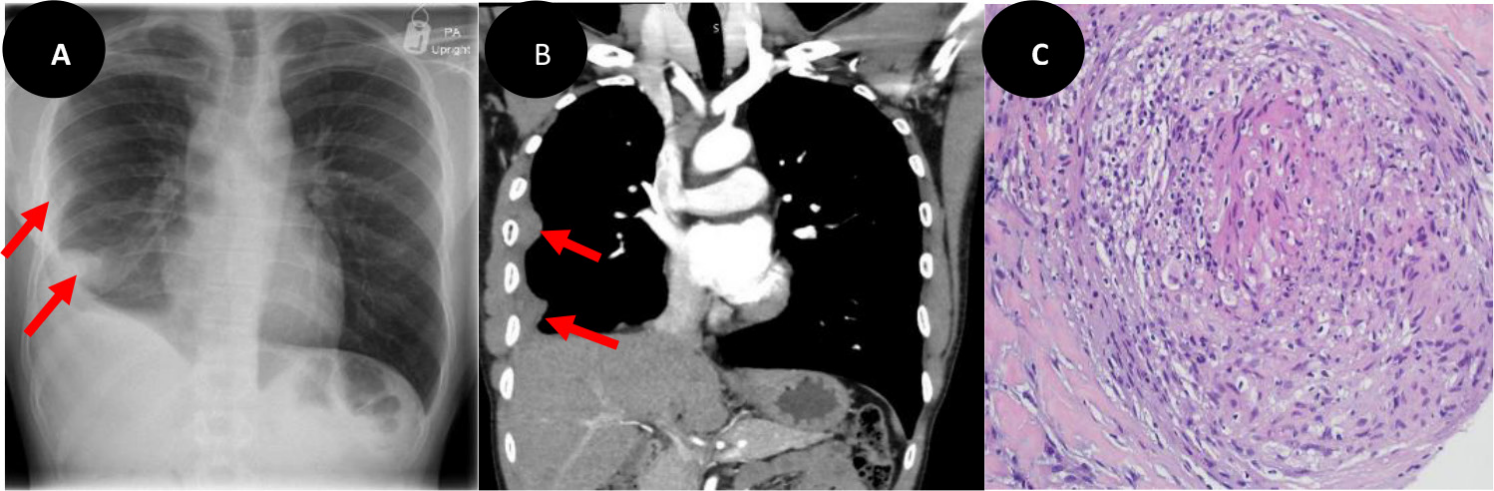
A chest radiograph shows right lower & middle zones pleural based nodular opacifications (red arrows) (A). A coronal CT chest view demonstrates nodular pleural thickening of the right lower and middle lobes (red arrows) and right hilar adenopathy (B). A microscopic view of pleural biopsy shows necrotizing granulomatous inflammation (C).

A 34-year-old previously healthy male underwent routine immigration screening that revealed abnormal findings on a chest X-ray. The initial imaging indicated right-sided pleural-based opacities (Figure 1A), prompting further investigation with chest computed tomography (CT). The CT scan revealed nodular pleural thickening of the right lower and middle lobes and right hilar adenopathy with no other significant lung parenchymal abnormalities, raising concerns for malignancy (Figure 1B). A CT-guided pleural biopsy was conducted to establish a definitive diagnosis. Surprisingly, histopathological examination of the biopsy specimen revealed necrotizing granulomatous inflammation with a positive TB culture, consistent with tuberculosis and deviating from the anticipated malignancy (Figure 1C).

## Conclusion

The atypical presentation observed in this patient emphasizes the diverse manifestations of TB that can mimic malignancy. This case highlights the significance of considering TB in the differential diagnosis of nodular pleural thickening, even in the absence of classical clinical and radiological features, allowing for early recognition that is vital for timely diagnosis and treatment.

## Conflict of Interest

The authors declare that they have no known competing financial or personal interests.
